# Exacerbation of Autoimmune Neuro-Inflammation in Mice Cured from Blood-Stage *Plasmodium berghei* Infection

**DOI:** 10.1371/journal.pone.0110739

**Published:** 2014-10-17

**Authors:** Rodolfo Thomé, André Luis Bombeiro, Luidy Kazuo Issayama, Catarina Rapôso, Stefanie Costa Pinto Lopes, Thiago Alves da Costa, Rosária Di Gangi, Isadora Tassinari Ferreira, Ana Leda Figueiredo Longhini, Alexandre Leite Rodrigues Oliveira, Maria Alice da Cruz Höfling, Fábio Trindade Maranhão Costa, Liana Verinaud

**Affiliations:** 1 Department of Structural and Functional Biology, Institute of Biology, University of Campinas, Campinas, Brazil; 2 Department of Histology and Embryology, Institute of Biology, University of Campinas, Campinas, Brazil; 3 Department of Genetics, Evolution and Bioagents, Institute of Biology, University of Campinas, Campinas, Brazil; 4 Department of Hematology, Faculdade de Ciências Médicas, University of Campinas, Campinas, Brazil; Instituto de Ciências Biomédicas/Universidade de São Paulo – USP, Brazil

## Abstract

The thymus plays an important role shaping the T cell repertoire in the periphery, partly, through the elimination of inflammatory auto-reactive cells. It has been shown that, during *Plasmodium berghei* infection, the thymus is rendered atrophic by the premature egress of CD4^+^CD8^+^ double-positive (DP) T cells to the periphery. To investigate whether autoimmune diseases are affected after *Plasmodium berghei* NK65 infection, we immunized C57BL/6 mice, which was previously infected with *P.berghei* NK65 and treated with chloroquine (CQ), with MOG_35–55_ peptide and the clinical course of Experimental Autoimmune Encephalomyelitis (EAE) was evaluated. Our results showed that NK65+CQ+EAE mice developed a more severe disease than control EAE mice. The same pattern of disease severity was observed in MOG_35–55_-immunized mice after adoptive transfer of *P.berghei*-elicited splenic DP-T cells. The higher frequency of IL-17^+^- and IFN-γ^+^-producing DP lymphocytes in the Central Nervous System of these mice suggests that immature lymphocytes contribute to disease worsening. To our knowledge, this is the first study to integrate the possible relationship between malaria and multiple sclerosis through the contribution of the thymus. Notwithstanding, further studies must be conducted to assert the relevance of malaria-induced thymic atrophy in the susceptibility and clinical course of other inflammatory autoimmune diseases.

## Introduction

The thymus plays a crucial role in the development and maturation of T lymphocytes. Inside the organ, thymocytes (T cells in the thymus) are provided with a wealthy microenvironment consisting of cytokines, chemokines and cell-cell interactions that ultimately leads to proliferation, T-cell receptor gene rearrangements and thymocyte differentiation into mature T cells [1]. However, the thymus undergoes weight and cellular loss caused by ageing and environmental disturbances, such as infections and malnutrition [2]. We have previously shown that infection with *Plasmodium berghei* NK65 infection, the non-cerebral malaria pathological agent, renders the thymus atrophic through the enhanced thymocyte death by apoptosis and premature egress of CD4^+^CD8^+^ (Double-positive, DP) T cells to the periphery [3]–[5].

It is already known that some viral and bacterial infections can promote the development of autoimmunity by inducing the breakdown of T cell tolerance and development of effector T cells reactive with the self-antigens or by the phenomenon called molecular mimicry, where a foreign antigen shares sequence or structural similarities with self-antigens [6], [7]. For instance, acute rheumatic fever, where antibodies attack the heart, can occur after the body makes immune responses against Group A β-hemolytic streptococci [8], [9]. In addition, it has been proposed that the prematurely egressed DP-T cells observed during *Trypanosoma cruzi* infection play an important role in the autoimmune cardio-inflammation [10].

Experimental Autoimmune Encephalomyelitis is a T cell-driven inflammation of the Central Nervous System (CNS) that presents similar characteristics to human Multiple Sclerosis [11]. In this model, following an inflammatory stimulus containing neuro-peptides T cells migrate from the peripheral immune system towards the CNS where they promote inflammation through the release of inflammatory mediators such as cytokines and chemokines [12], [13]. Cells from the Th1 and Th17 subsets are important for disease establishment, as evidenced by previous reports [14]–[16]. Both in the human and animal diseases T cells play a major role. Therefore, changes in the subpopulations of T cells influence the outcome and susceptibility to autoimmune development.

In this context, we aimed to evaluate whether the previous infection with *Plasmodium berghei* NK65 would interfere with the clinical course of Experimental Autoimmune Encephalomyelitis, a mouse model for human Multiple Sclerosis (MS). We observed that EAE-susceptible mice cured from malaria developed an aggravated form of EAE, with increased infiltration of DP-T cells in the Central Nervous System (CNS). Further analyses showed that thymic-prematurely egressed DP-T cells were important for the enhanced clinical manifestation of the disease. To our knowledge, this is the first study to demonstrate the possible integration between malaria and EAE through the contribution of the thymus.

## Materials and Methods

### Animals

Six- to eight-week-old female C57BL/6 mice from the Multidisciplinary Center for Biological Research, University of Campinas, were used in this study. Mice were kept in specific-pathogen free conditions, in a controlled temperature and photoperiod environment, with free access to autoclaved food and water throughout the experiment. All protocols involving laboratory animals were approved and performed in accordance with the guidelines of the Institutional Committee on the Use and Care of Animals (CEUA, #2687–1).

### Infection and treatment

For these experiments, we used the NK65 strain of *Plasmodium berghei*, because this parasite do not accumulate in the CNS of mice to cause cerebral malaria. Mice (n = 6 mice/group) were intra-peritoneally injected with 10^6^ *Plasmodium berghei*-infected red blood cells (iRBCs) obtained from a source mouse. The frequency of iRBCs was assessed daily by examination with Giemsa-stained thin blood smear. Ten days after infection, animals of each group started the treatment with chloroquine (5 mg.kg^−1^, via i.p. for five consecutive days). Three days after the last dose of the drug, mice were immunized for the induction of EAE.

### EAE induction and evaluation

EAE was induced and evaluated in mice according to a previous published paper [17]. Briefly, each mouse was subcutaneously injected with 100 µg MOG_35–55_ (MEVGWYRSPFSRVVHLYRNGK, Genemed Syn, USA) emulsified with Complete Freunds Adjuvant (CFA, Sigma-Aldrich, USA). 200 ηg Pertussis toxin (Ptx, Sigma-Aldrich, USA) was administrated via i.p. at 0 and 48 h after MOG_35–55_ inoculation. Clinical signs were followed and graded daily according to a score method, where 0: no sign, 1: flaccid tail, 2: hind limbs weakness, 3: hind limbs paralysis, 4: hind paralysis and fore limbs weakness, 5: full paralysis/dead. Disease severity was evaluated for thirty days. At the end of the experiment, results were analyzed by linear regression, thinner lines indicate 95% confidence interval.

### Determination of the thymic index

The calculations to determine the thymic index were previously described [3]. Briefly, at the indicated time-points, the gross weight of each mouse was recorded and then the thymuses were collected and weighed. The thymic index was calculated using the formula: organ weight (g)/body weight (g) ×100.

### Cell sorting and flow cytometry

Spleen cells were obtained from *P.berghei*-infected mice at ten days of infection. Total splenic T lymphocytes were isolated using Dynabeads following the manufactureŕs instructions (Mouse Pan T cell isolation kit, Life Technologies, USA). For DP-T cell isolation, spleen-derived single cell suspension was incubated with FITC-conjugated anti-CD4 (clone H129.19) and PE-conjugated anti-CD8a (clone 53–6.7) for 20 minutes. CD4^+^, CD8^+^ and CD4^+^CD8^+^ (double-positive) T lymphocytes from malaria-bearing mice at ten days of infection were sorted in FACSAria II cell sorter (BD Biosciences, USA). The purity of isolations was assessed and accounted for 98% pure subpopulations. For adoptive transfer experiments, 1,5×10^5^ cells were adoptively transferred (via i.v.) to each group of mice at the onset of EAE (around the tenth day after immunization). For activation experiments, sorted cells were seeded in U-bottom 96 well/plates (2×10^4^ cells/well) and incubated for 72h with lipopolysaccharide (1 ng/mL, from *E. coli*, Sigma-Aldrich, USA), *Plasmodium berghei* extracts (PbX, 50 µg/mL) or MOG_35–55_ peptide (20 µg/mL, Genscript, USA). At the end of culture period, the supernatants were collected and assayed for detection of mouse IFN-γ and IL-17 by Cytometric Bead Assay (CBA, BD Biosciences, USA). In some set of experiments, total T cells isolated from naïve and malaria-bearing mice were transferred (1x10^6^ cells/mouse) at the onset of EAE.

### Preparation of Plasmodium berghei extracts

The production of extracts from *P. berghei*-infected RBCs (iRBC) followed a previously published recommendation [18]. Briefly, iRBC-enriched suspension was submitted to 20 cycles of freeze/thawing in liquid nitrogen and warm bath (37°C). The protein concentration was determined using the Bradford Protein Assay following the manufactureŕs instructions (Sigma-Aldrich, St Louis, MO, USA).

### Analysis of cellular infiltration in the CNS

Fourteen days after EAE induction, mice were anesthetized, perfused with ice cold PBS and half of the spinal cords were removed and stored at −80°C until use for RT-PCR assays; the remaining tissue was prepared for the enrichment of infiltrating leukocytes according to a previously described methodology and analyzed by flow cytometry [34].

### RT-PCR assays

Mice were killed at the indicated time-points and frozen tissues were used for RNA extraction (Trizol reagent, Life Technologies, USA) and cDNA synthesis according to the manufactureŕs recommendations (High Capacity RNA-to-cDNA converter kit, Life Techcnologies, USA). RNA extraction from DP-T cells was carried our immediately after sorting, using an extraction kit according to the manufactureŕs instruction (RNeasy Micro kit, Qiagen USA). Expression of AIRE (Mm00477461_m1), IL-7 (Mm01295803_m1), IL-6 (Mm00446190_m1), IL-17 (Mm00439618_m1), IFNg (Mm01168134_m1), FOXP3 (Mm00475162_m1) and RAR-related orphan receptor C (RORc) (Mm01261022_m1) were analyzed in comparison to GAPDH (Mm99999915_g1, housekeeping gene) levels. RT-PCR reactions were performed using Taqman reagents according to manufactureŕs recommendations (Applied Biosystems, USA). Expression levels of genes were represented as a relative copy numbers by using the method of delta threshold (2^−ΔΔCt^).

### Histopathology and immunofluorescence

At the indicated time-points mice were killed, spinal cords were removed and snap frozen; 12 µm thin slices were made in cryostat and stained with haematoxylin and eosin (H&E) for histopathological analysis. For the characterization of the infiltrating cells, the reactive sites of the slices were blocked with Phosphate-Buffer (0,1M, pH7,2)-BSA 3%. The cells were stained with FITC-conjugated rat anti-mouse CD4 (clone H129.19, at a 1:100 dilution, BD Biosciences, USA) and PE-conjugated rat anti-mouse CD8a (clone 53–6.7, at a 1:100 dilution, BD Biosciences, USA). In some set of experiments, the lumbar spinal cords were incubated with purified anti-mouse GFAP, COX-2, IL-6, NF-κB, phosphorylated iKB and iNOS. Later, secondary Cy3- or TRITC-conjugated antibodies were added. The reaction was analyzed under epifluorescence microscope (Leica, GER).

### *In vitro* re-stimulation and cytokine dosage

Splenic cells were aseptically collected from mice after 10 days of MOG_35–55_ immunization. Single cell suspensions were stained with Carboxyfluorescein succinimidyl ester (CFSE, 2,5 µM, Sigma-Aldrich, USA) following the manufactureŕs instructions. Cells (5×10^5^/well) were diluted in RPMI 1640 media supplemented with Fetal Calf Serum (FCS; 10% vol/vol), guaramicine (50 µg/mL), 2-Mercaptoethanol (2 mM) and myelin oligodendrocyte glycoprotein peptide (MOG_35–55_; 20 µg/mL), plated in U-bottom plates and incubated for 96 h. After the incubation period, cells were stained with PercPCy5-conjugated anti-CD3e, PE-conjugated anti-CD8a and PECy7-conjugated anti-CD4 antibodies and fixed in 1% paraformaldehyde prior to flow cytometer analysis. CFSE^low^ cells inside each population were considered proliferating T cells. Culture supernatants were collected and assayed for cytokines (IL-4, IL-6, IL-10, IL-17, IFN-γ and TNF-α) secretion using the Cytometric Bead Array (CBA, BD Biosciences, USA) according to manufactureŕs instructions.

### T cell co-cultures

For the co-cultures, total spleen T cells were isolated from malaria-bearing and EAE-inflicted mice after ten days of immunization. T cells derived from EAE mice were stained with CFSE according to the manufactureŕs instructions (2,5 µM, Sigma-Aldrich, USA) and seeded in U-bottom 96-well plates (5×10^5^ cells/well). T cells derived from naïve or *P.berghei*-infected mice were added afterwards at a 1∶1 proportion. MOG_35–55_ peptide was added to a final concentration of 20 µg/mL. The cultures were incubated for 96 h at 37°C. At the end of culture time, the cells were collected and stained with PercP-conjugated anti-CD3e antibodies and fixed in 1% paraformaldehyde prior to flow cytometer analysis. CFSE^low^CD3^+^ cells were considered proliferating responder T cells.

### Statistical analysis

Clinical score comparisons between control and experimental groups were done by Two-Way ANOVA and post-tested with Bonferroni. Other analyses among two and three (or more) groups were carried out with Students t test and One-Way ANOVA, respectively. Results are expressed as mean ± standard error mean (SEM) and p<0,05 value were defined as significant.

## Results

### Exacerbation of Experimental Autoimmune Encephalomyelitis in mice cured from *P.berghei* infection

**Figure 1:**
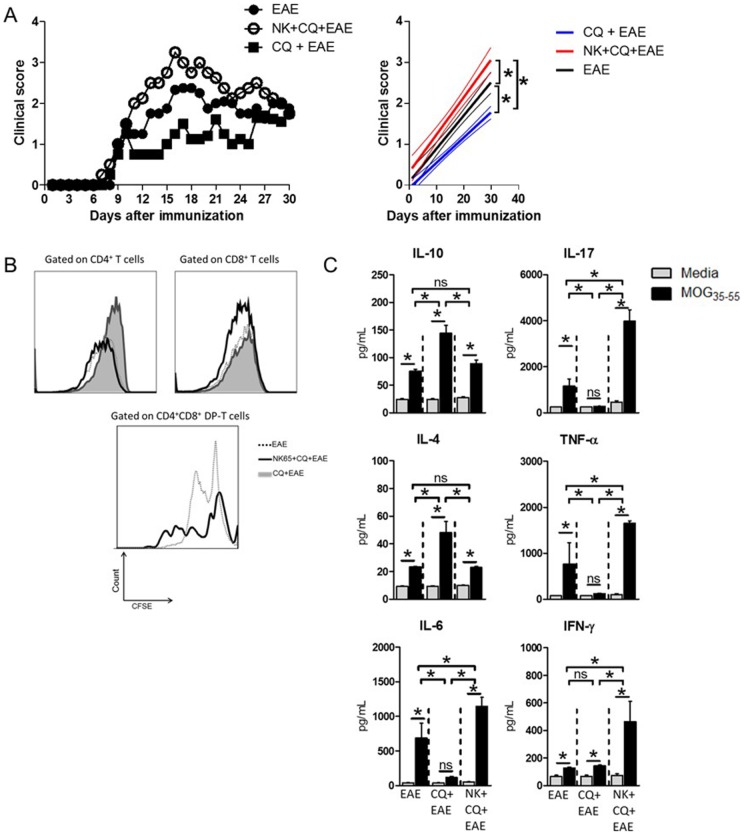
Aggravation of EAE in mice cured from malaria correlates with increased cellular immune response towards myelin. C57BL/6 mice (n = 6 mice/group) were intraperitoneally (i.p.) infected with 1×10^6^ *P.berghei*-infected Red Blood Cells and treated with chloroquine (CQ, 5 mg/Kg) for five consecutive days starting at the 10^th^ day after infection. Three days after the last dose of CQ, mice were immunized with 100 µg of MOG_35–55_ peptide and Pertussis toxin was administrated (via i.p.) at 0 and 48h after peptide immunization for EAE induction. A) The clinical course of EAE was then monitored. Linear regression analyses are exposed in the side panels, thinner lines indicate 95% confidence interval. B) At the 10^th^ day after MOG-immunization, the spleens of mice were collected and dissociated. Total leukocytes (5×10^5^/well) were CFSE-stained (2,5 µM) and cultured in the presence of MOG_35–55_ (10µg/mL) peptide for 96h. At the end of culture period, the cells were surface stained with anti-CD3/CD4/CD8 antibody cocktail and events were acquired in a flow cytometer. The proliferation was analyzed inside each T cell population. C) The culture supernatants were assayed for the secreted cytokines IL-10, IL-4, IL-6, IL-17, TNF-α and IFN-γ. Data was analyzed by One-Way Anova and post-tested with Bonferroni. In all analyses, *: p<0,05; ns: not significant. Representative data of three independent experiments.

The present study aimed to evaluate whether the previous infection with *P.berghei* NK65 would interfere with the clinical outcome of Experimental Autoimmune Encephalomyelitis. For that purpose, C57BL/6 mice were infected with parasitized erythrocytes and at the tenth day of infection, parasites were eliminated following chloroquine (CQ) treatment. Three days after the last dose of CQ, mice were immunized with MOG_35–55_ peptide for the induction of EAE (NK+CQ+EAE group). As a control, besides EAE-bearing mice (EAE group), we used naïve mice injected with non-parasitized erythrocytes and treated with chloroquine before EAE induction (CQ+EAE group). The results showed that mice from the NK+CQ+EAE group developed an aggravated disease course with higher clinical score when compared to EAE group (Figure 1A). Also noteworthy is the mild clinical scores developed by mice from CQ+EAE group. As recently published by our group, CQ reduces the severity of EAE probably by induction of regulatory T cells [17]. Thus, our results also show that *P.berghei* infection was able to overcome the suppressive effect of chloroquine.

### The aggravation of EAE correlates with DP-T lymphocytes reactivity to neuro-peptides

Since EAE model is highly dependent on the cellular response against neuro-antigens, the cellular immune response against MOG peptide was evaluated. Spleen cells from EAE-bearing mice were harvested ten days after neuro-peptide immunization and cultivated in the presence of MOG_35–55_. The data obtained showed that NK+CQ+EAE-derived cells proliferated significantly more when compared to cells from EAE control group (Figure 1B). Among the proliferating cells, the supopulation of DP-T cells had the most active proliferation, while CQ+EAE mice presented no DP-T cells in the periphery. Cells from CQ+EAE group did not present significant proliferation or the presence of DP-T cells in the spleens (Figure 1B). The cytokine secretion was altered as well. Cell culture supernatants from NK+CQ+EAE mice presented significant higher levels of pro-inflammatory cytokines (TNF-α, IL-17 and IFN-γ) compared with the EAE group, whereas the levels of IL-10, IL-6 and IL-4 remained similar between the two groups (Figure 1C). Interestingly, cell cultures from CQ+EAE mice showed significantly higher levels of IL-10 and IL-4, and lower levels of IL-17, IL-6 and TNF-α than the other two groups (Figure 1C).

### Plasmodium berghei NK65 infection promotes thymic alterations and the premature egress of CD4^+^CD8^+^ double-positive lymphocytes to the periphery

**Figure 2:**
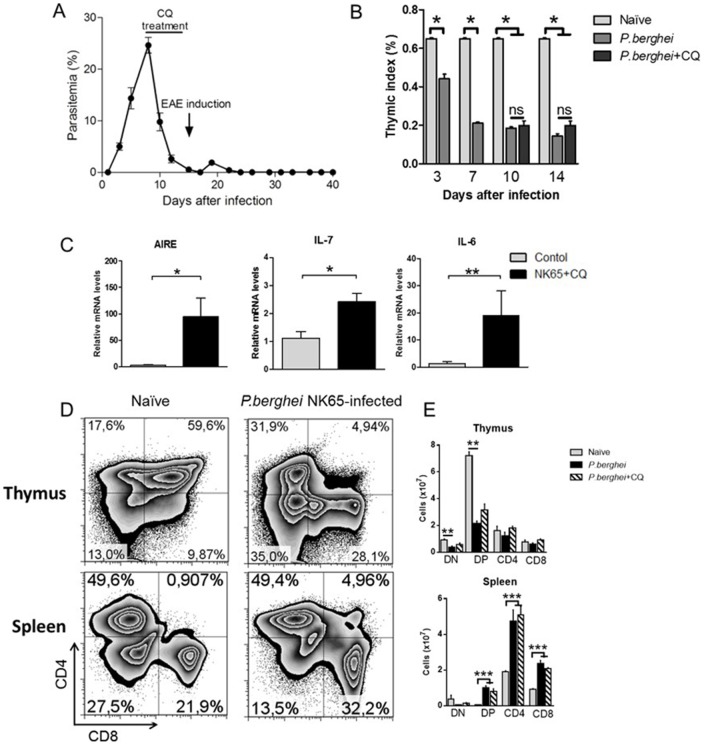
*P. berghei* infection provokes thymic alterations and the premature egress of double-positive T lymphocytes in the spleens. C57BL/6 mice (n = 6 mice/group) were intraperitoneally (i.p.) infected with 1x10^6^ *P. berghei*-infected Red Blood Cells and treated with chloroquine (CQ, 5mg/Kg) for five consecutive days starting at the 10^th^ day after infection. A) Mice treated with CQ showed decrease in the parasite burden. B) The thymuses were collected at different time-points and the relative body weight was determined. C) The gene expression of AIRE, IL-7 and IL-6 was assessed in the thymuses of *P. berghei* NK65-infected mice at the fifteenth day of infection. Flow cytometry analysis of the frequency (in D) and absolute numbers (in E) of T lymphocytes subpopulations (CD4^+^ and CD8^+^) in the thymuses and spleens. Data was analyzed by Student's t test. In all analyses, *: p<0,05 and **: p<0,01. Ns: not significant. #: p<0,05 in comparison with untreated mice. Representative data of four independent experiments.

The results presented so far show that the exacerbated clinical score from NK+CQ+EAE mice correlates with an enhanced cellular immune response towards neuro-antigens orchestrated, partly, by DP-T cells. The presence of DP-T cells in the spleens of NK+CQ+EAE mice is interesting. It was previously shown that during *P.berghei* infection, the thymus of BALB/c mice undergoes structural and phenotypical changes that together with the uncontrolled parasitemia culminates in death of the host fourteen days after infection [3]. To investigate whether the infection with *P.berghei* would promote similar alterations in the spleens and thymuses of a different mouse strain, C57BL/6 (B6) mice were injected with *Plasmodium berghei*-infected Red Blood Cells. In this strain, the infection is controlled when chloroquine (5 mg.kg^−1^) is administrated for five consecutive days starting at day 10 after infection (Figure 2A). Similarly as in the BALB/c model, the thymus of B6 mice is reduced with loss of the thymic index, starting at the third day of infection (Figure 2B). Interestingly, the expression of the Autoimmune Regulator (AIRE) gene was found up-regulated compared with control mice as well as the expression of IL-7 and IL-6 (Figure 2C). The gene expression within the thymus of B6 mice infected with other *Plasmodium* species, such as *P.yoelli* and *P.chabaudi* was also evaluated and the results showed that the gene expression of cytokines and AIRE differs between groups (Figure S1).

It is already clear nowadays that disarranged thymic structure associated with an inflamed microenvironment result in altered maturation of thymocytes [19]. Accordingly, the thymocyte subpopulations were altered in the thymus of *P.berghei*-infected mice, with reduced numbers of CD4^+^CD8^+^ (Double-positive, DP) and increased frequency of CD4^+^ and CD8^+^ thymocytes (Figure 2D). In the spleen, results showed an increased frequency of DP-T lymphocytes as well as CD4^+^ and CD8^+^ T cells. Naïve mice treated with chloroquine showed no alterations regarding the frequency of DP-T cells in the spleen and in thymic T cell subpopulations in comparison to mice without treatment (*data not shown*). Thus, it is possible to suppose that the responding DP-T cells observed in the NK+CQ+EAE mice was elicited during *P.berghei* NK65 infection and that these cells are related to the exacerbated EAE outcome.

### Infiltration of inflammatory cells in the central nervous system is characterized by the presence of DP-T cells and the local production of inflammatory cytokines

**Figure 3:**
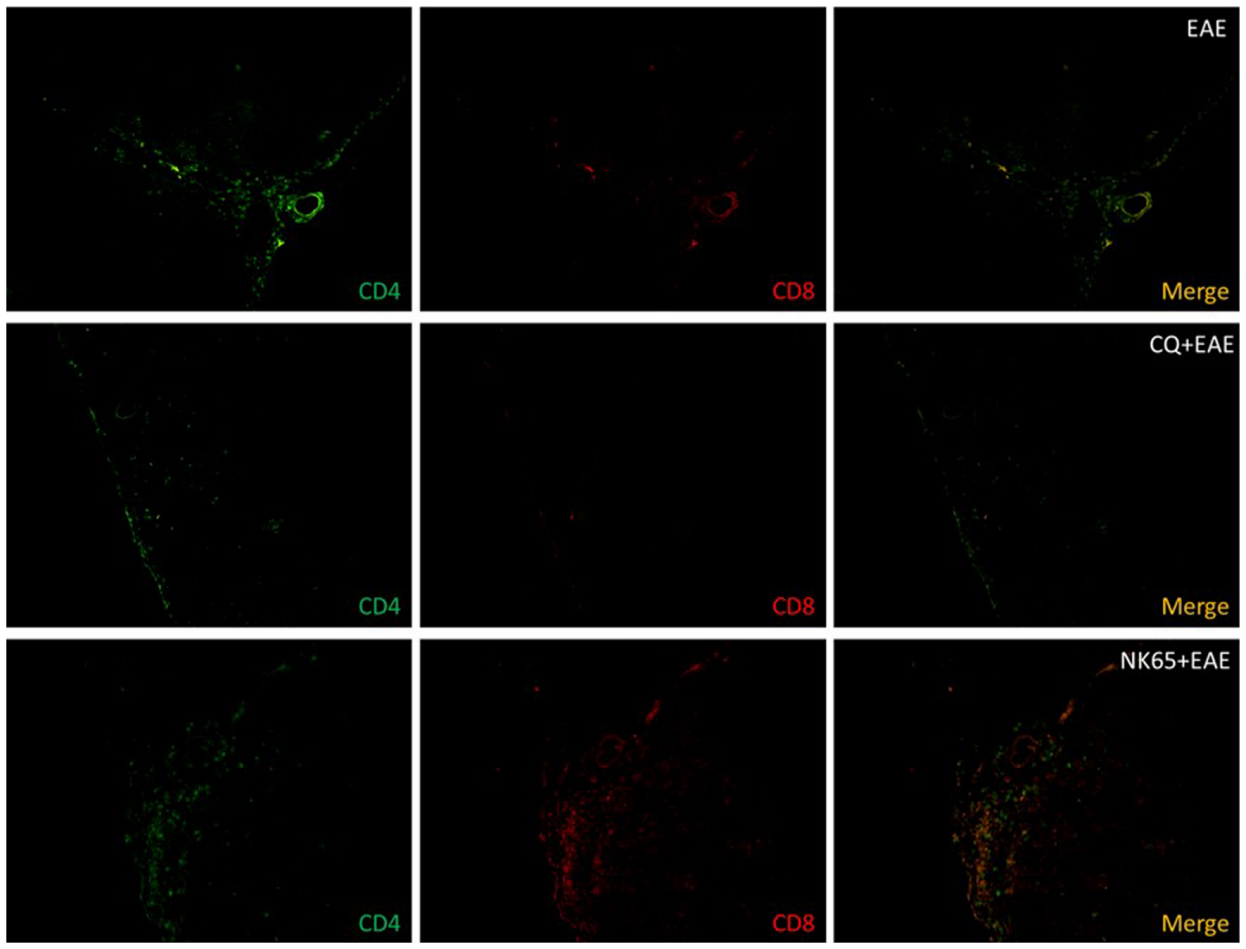
Central Nervous System of malaria-cured EAE mice show increased cellular infiltration of DP-T cells. C57BL/6 mice (n = 6 mice/group) were intraperitoneally (i.p.) infected with 1×10^6^ *P.berghei*-infected Red Blood Cells and treated with chloroquine (CQ, 5 mg/Kg) for five consecutive days starting at the 10^th^ day after infection. Three days after the last dose of CQ, EAE was induced. As controls, naïve mice were treated with CQ or vehicle before EAE induction. The spinal cords of EAE-inflicted mice were collected fourteen days after MOG-immunization. Frozen thin sections (12 µm) were made and fixed in formalin. Cells were stained with FITC-conjugated anti-CD4 and PE-conjugated anti-CD8 and analyzed in epifluorescence microscope. Figures are representative of three independent experiments. Magnification: 200X.

To investigate the possible association of DP-T cells with the exacerbation of EAE, the frequency of these cells in the Central Nervous System was evaluated. Fourteen days after EAE induction, mice were killed and spinal cords were prepared for histological analyses. The phenotype of the infiltrating T cells was determined by direct immunofluorescence labeling technique using FITC-conjugated anti-CD4 and PE-conjugated anti-CD8 antibodies. Results showed that in EAE group, among the infiltrating cells, a high frequency of CD4^+^ T cells was observed, while no infiltration was observed in the CNS of CQ+EAE mice (Figure 3). As expected, an elevated frequency of CD4^+^CD8^+^ (DP) T cells among the infiltrating cells in the CNS of NK+CQ+EAE mice was identified (Figure 3). These data indicate that the double-positive T cells that prematurely migrated from the thymus during *P.berghei* infection are antigen-responsive (Figure 1B) and capable to migrate to the CNS during neuro-inflammation (Figure 3). In addition, the CNS of NK+CQ+EAE mice showed an increased production of IL-6, NF-κB, phosphorylated iκB (iκBα-p), iNOS, COX-2 and GFAP compared with EAE group (Figure S2).

**Figure 4:**
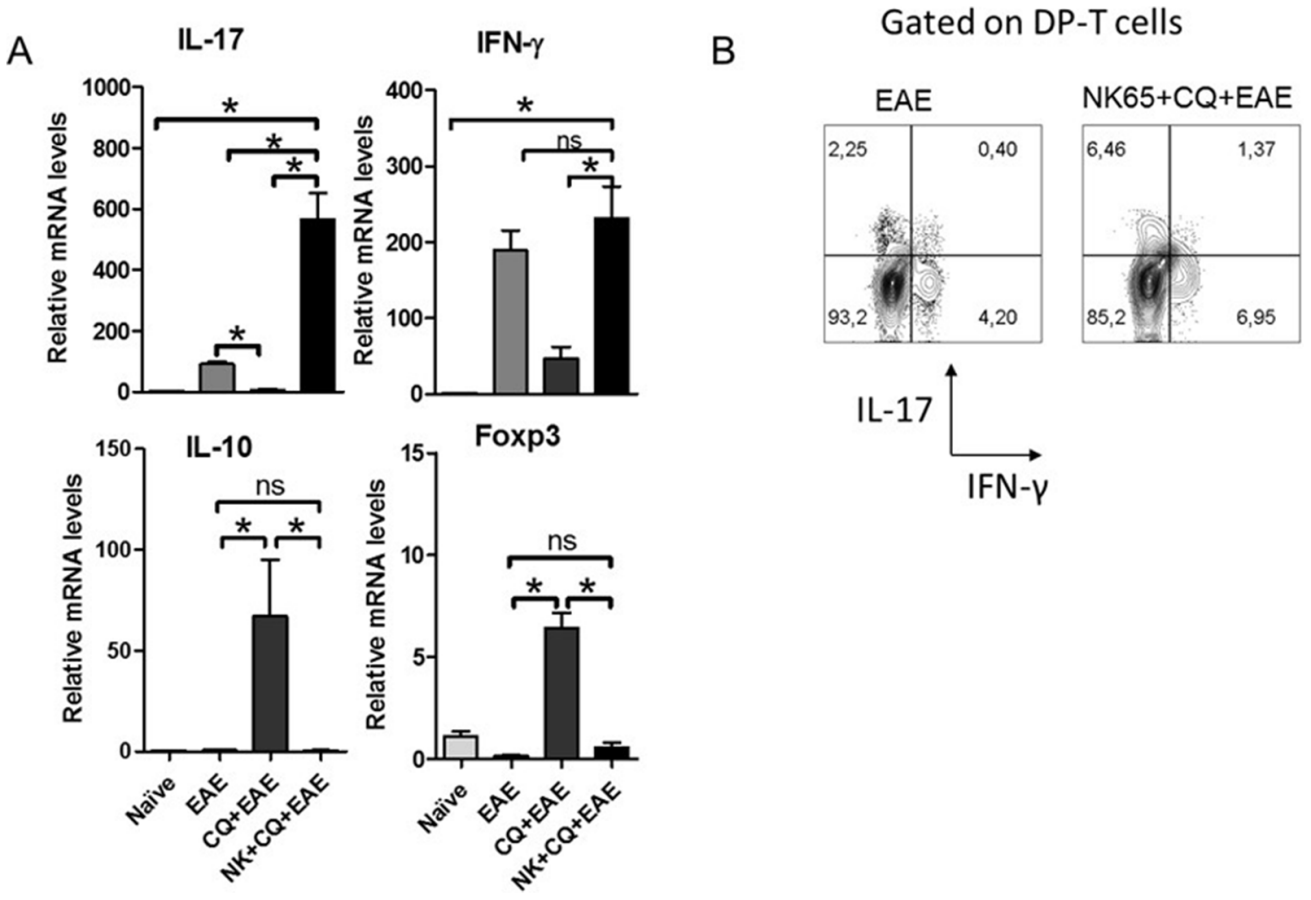
Inflammation in the CNS of NK+CQ+EAE mice correlates with an increased production of inflammatory cytokines by DP-T cells. Groups of mice (n = 6 mice/group) subjected to infection and EAE induction. A) At the 10^th^ day after MOG-immunization, mice were killed and spinal cords were removed to analyze the gene expression of IL-17, IFN-γ, Foxp3 and IL-10 in the lumbar spinal cords of mice. Data was analyzed by One-Way Anova and post-tested with Bonferroni. B) The infiltrating cells of the CNS were enriched and stimulated by Phorbol Myristate Acetate and Ionomycin in the presence of Brefeldin A for 4 h. The frequency of IFN-γ- and IL-17-producing cells inside CD4^+^CD8^+^ T cell gate was analyzed. In all analyses, *: p<0,05. ns: not significant. Representative data of three independent experiments.

In the CNS, an increased gene expression of IL-17 in tissue from NK+CQ+EAE mice was observed in comparison with mice from EAE group (Figure 4A). In contrast, the expression of Foxp3 and IL-10 was not significantly altered in comparison with the naïve group (Figure 4A). IFN-γ expression was not significantly altered. To investigate whether DP-T cells found in the CNS tissue were able to produce inflammatory mediators, mice were killed at the fourteenth day of EAE and the infiltrating cells were enriched from the CNS. The intracellular staining results showed that NK+CQ+EAE mice had higher frequency of IFN-γ- and IL-17-producing DP-T cells than EAE mice. (Figure 4B).

### Transfer of lymphocytes from *P.berghei*-infected mice increases the severity of EAE

**Figure 5:**
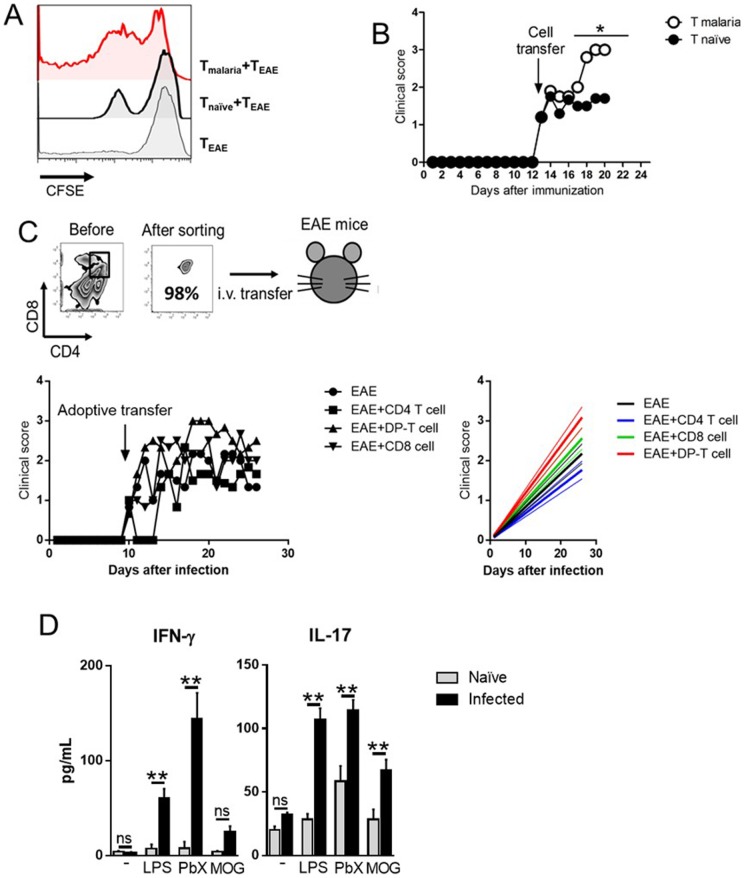
Exacerbation of EAE in mice transferred with total T from naïve mouse or DP-T cells from malaria-cured mouse. A) C57BL/6 mice (n = 6) were intraperitoneally (i.p.) infected with 1×10^6^ *P.berghei*-infected Red Blood Cells. At the 10^th^ day of infection, splenocytes were collected and total T cells were isolated using dynabeads (Pan T cell isolation kit). Naïve-derived splenic T cells were used as well. EAE-inflicted mice (n = 6) were killed at the 10^th^ day after immunization and the total splenic T cell were isolated with the same methodology and CFSE-stained (2,5 µM). As controls, T cells from EAE-inflicted mice were cultured without the presence of other cells. B) Total T cells isolated from naïve and malaria-bearing mice were transferred (1×10^6^ cells/mouse) at the onset of EAE (n = 6 mice/group), and clinical course of the disease was evaluated daily. Data was analyzed by Two-Way ANOVA and post-tested with Bonferroni. C) Sorted splenic CD4^+^, CD8^+^ and CD4^+^CD8^+^ T cells from malaria-bearing mice were adoptively transferred (1,5×10^5^ cells/mouse) to EAE-inflicted mice (n = 6 mice/group) at disease onset. The clinical score of the disease was evaluated daily. The side panel contains the linear regression lines with the 95% confidence interval (thinner lines). D) Malaria-bearing mice were killed at 10 days after infection and the DP-T cells were sorted and incubated with LPS (1 ng/mL), *P. berghei* extracts (50 µg/mL) and MOG_35-55_ peptide (20 µg/mL) for 72 h, at the end of culture period the supernatants were removed and assayed for the detection of IFN-γ and IL-17. Analyses were conducted using Student's t test. *: p<0,05. **: p<0,01. Representative data of two independent experiments with similar results.

In order to better characterize the relationship among malaria-elicited DP-T lymphocytes and the exacerbation of EAE, a series of experiments to demonstrate that the mechanism of disease aggravation is T-cell dependent was conducted. First, splenic T cells isolated from *P.berghei*-infected or naïve mice were co-cultured with splenic T cells from EAE-bearing mice in the presence of MOG_35–55_ peptide for four days. Results showed that the cultures conducted in the presence of T cells from malaria-infected mice proliferated significantly more than cells from co-cultures with naïve T cells (Figure 5A).

Then, it was investigated whether the adoptive transfer of splenic T cells from malaria mice would alter the course of EAE. For that purpose, B6 mice were infected with *Plasmodium berghei* NK65 and the total splenic T cells were isolated ten days after infection. These cells were adoptively transferred to EAE mice at the beginning of the clinical signs (around the 10^th^ day after immunization). As expected, the clinical course of EAE was significantly aggravated in malaria T cells-transferred mice compared to naïve T cell-recipient mice (Figure 5B).

Lastly, since the infiltrating T cells in the CNS of NK+CQ+EAE mice comprised mostly of DP-T cells, the next goal was to evaluate whether these cells play a role in the aggravation of EAE. Therefore, sorted splenic CD4^+^, CD8^+^ and DP-T cells from malaria-infected mice were intravenously transferred to EAE mice as soon as the clinical signs of EAE started to appear (around the 10^th^ day after MOG_35–55_ immunization). The results showed that mice that received DP-T cells developed a more severe disease than mice that received CD4^+^ and CD8^+^ T cells (Figure 5C). In addition, we aimed to evaluate whether the cellular response elicited by DP-T cells were antigen-specific or were raised in a by-stander fashion. For that purpose, we sorted DP-T cells from the spleens of naïve and *P. berghei*-infected mice and cultivated the cells in the presence of LPS, *P. berghei* extracts and MOG peptide. At the end of the culture time, the supernatants were assayed for the presence of inflammatory cytokines. As depicted in figure 5D, cultures conducted in the presence of LPS and PbX produced elevated levels of IFN-γ and IL-17 in comparison with DP-T cells from naïve mice. Interestingly, the presence of MOG was able to stimulate the production of IL-17, but not IFN-γ, in DP-T cell cultures. This data shows that the prematurely egressed DP-T cells from *P. berghei*-infected mice are able to produce inflammatory cytokines in the presence of PAMPs (LPS), specific stimulus (PbX) and auto-antigens (MOG).

## Discussion

In this study, we show that, after infection with *Plasmodium berghei* NK 65, MOG-immunized mice develop an aggravated form of Experimental Autoimmune Encephalomyelitis. Further analysis demonstrated that the thymic prematurely egressed CD4^+^CD8^+^ (double-positive) T cells play an important role in the enhanced severity of the disease.

The thymus is a key organ for the development of T lymphocytes and plays a major role in the elimination of auto-reactive T cells [20], [21]. T cell precursors enter the thymus through the high endothelial venules found in the cortico-medullary junction and lack the expression of antigen receptor and co-receptors CD4 and CD8 [22]. The maturation process consists of both migration of thymocytes through the cortex towards the medulla and expression of T cell receptor and co-receptors [23]. During this process, immature T cells interact with a myriad of cytokines, chemokines and self-peptides presented in association with MHC molecules on thymic epithelial cells (TECs) and cells that recognize antigens with high avidity are deleted [19]. We have previously shown that the lethal *Plasmodium berghei* NK65 infection promotes thymic atrophy and that this phenomenon is dependent on thymocyte death and its premature egress to the periphery [3]–[5]. The lymphocytes subpopulations was altered in the thymus as well, with increased frequency of simple positive CD4^+^ and CD8^+^ T cells. However, due to the loss of thymic mass, the absolute numbers of cells were reduced. In the spleens, the augmentation in the numbers of CD4 and CD8 T cells may be explained by the clonal expansion observed in the course of malaria [24]–[26]. In addition, we observed an increased expression of AIRE, IL-6 and IL-7 in thymuses from *P.berghei*-infected mice, which indicate that an altered repertoire selection might be occurring. An impaired T cell maturation and selection in the thymus may lead to the release of self-reactive T cells to the periphery and the sub-sequential development of autoimmune disorders [27]–[29].

Indeed, we observed that mice, which were cured from *P.berghei* infection, developed a more severe form of EAE compared with uninfected animals. When we analyzed the inflammatory infiltration in the CNS, we found that most of the infiltrating cells were double-positive T cells. These DP-T cells were functional as they produced high levels of IL-17, an inflammatory cytokine related to EAE worsening [14], [30], [31]. As expected, when we adoptively transferred total T cells or sorted DP-T cells derived from malaria-bearing mice towards EAE-inflicted mice, the clinical score arose significantly. Taken together, these set of experiments demonstrated that the DP-T cells elicited in *P.berghei*-infected mice are inflammatory and exacerbate autoimmune neuro-inflammation. Co-cultures showed that malaria-elicited T cells stimulated the proliferation of T cells from MOG-immunized mice. Together with the proliferative response observed in DP-T cells from NK+CQ+EAE, these data suggest that DP-T cells, originated in the course of malaria, exacerbate EAE by, at least, two different mechanisms: (i) DP-T cells self-react and proliferate against neuro-antigens and (ii) DP-T cells stimulate the proliferation of encephalitogenic T cells. Of note, when cultured in the presence of different stimuli, DP-T cells in the spleens produced inflammatory cytokines IFN-γ and IL-17 at high levels. These observations indicate that DP-T cells elicited in *P. berghei* infection respond to Pathogen-Associated Molecular Patterns and specific antigens as well as to unrelated auto-antigens.

Interestingly, infection overcame the suppressive effect of chloroquine. We previously observed that CQ treatment reduces the severity of Experimental Autoimmune Encephalomyelitis through the expansion of regulatory T cells [17]. Later, our results showed that CQ is able to modulate dendritic cells *in vitro* directing them towards a tolerogenic profile that can be used to treat EAE [32]. The mechanisms by which *P. berghei* surpasses the CQ suppressive effect remains to be elucidated. Still, the recently described effect of *Plasmodium* infection in Toll-like receptors (TLR) priming may explain, at least in part, the aggravation of inflammation [33], [34].

The data presented here might be concerning as malaria remains the worlds most prevalent infectious disease, with over 250 million people infected [35]. Several reports have correlated autoimmunity prevalence in countries that have eradicated the *Plasmodium* infection [36]. Although the hygiene hypothesis supports the idea of infection-induced autoimmune resistance, this theory may not fully apply to malaria. There is an increasing amount of evidence supporting or rejecting the role of malaria in autoimmune disease both in human and animal models [37]–[47]. Similarly, it was previously proposed that in *Trypanosoma cruzi* infection, the inflammatory profile of prematurely egressed DP-T cells from the thymus may be involved in the autoimmune process observed in murine and human Chagas' disease [10].

It was demonstrated that *Plasmodium chabaudi* infection increases the frequency of regulatory T cells in spleens, and these cells suppressed the development of EAE in a by-stander fashion [48]. However, *P. berghei* infection was shown to induce the secretion of anti-DNA and anti-nuclear antibodies in a T cell-dependent manner [49]. These observations indicate that different *Plasmodium* species induce distinct types of immune responses. Indeed, we observed that the gene expression of inflammatory cytokines and AIRE within the thymus vary depending on the type of *Plasmodium* species. Nonetheless, infected Red Blood Cells from *P.falciparum*, *P.yoelli*, *P.berghei* and *P.vivax* induce distinct maturation status of dendritic cells (DCs) [18], [50]–[59]. Indeed, we have shown that DCs modulated with plasmodium extracts are able to reduce the severity of EAE through the suppression of inflammatory responses in the Central Nervous System [18].

Interestingly, it was recently observed that *Plasmodium chabaudi*-infected mice presented exacerbated response to bacterial challenge [60]. In this study, the authors showed that following infection, mice were hyper-responsive to *E. coli*-derived LPS in a mechanism that is dependent on NLRP12/NLRP3 inflammasome activation. The results presented by the authors are in line with others [33], [34], [61]. In our model, although we did not investigate the role of inflammasomes, we found that the T cell compartment is severely altered in *P. berghei*-infected mice resulting in an exacerbation of autoimmune responses. Still, further studies must be conducted in order to ascertain the possible interplay between innate cells-derived inflammasome and T cells in the hyper-responsive inflammation elicited by *P. berghei* infection.

**Figure 6:**
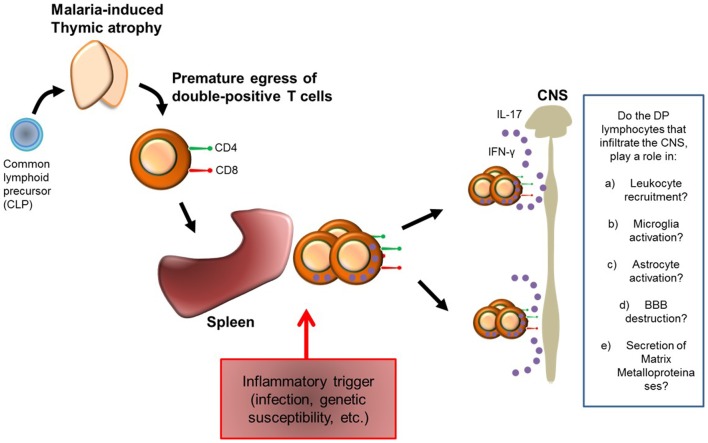
Hypothesis model for the exacerbation of autoimmune neuro-inflammation. Based in our observations, we propose a model for EAE exacerbation. Malaria infection promotes thymic atrophy and the premature egress of DP-T cells to the peripheral immune system. After an inflammatory trigger, which can be infection, genetic susceptibility or chronic inflammation, these cells proliferate and migrate to the target organ where they stimulate CNS inflammation by secreting cytokines. However, there is still much to be explored, as for example, whether these DP-T cells are able to induce leukocyte recruitment, microglia and astrocyte activation, and, Blood-Brain Barrier (BBB) destruction.

Based on our observations, we hypothesized that during malaria infection, thymocytes prematurely egress from the thymus towards the peripheral immune system. In the periphery, due to a trigger event (which can be genetic susceptibility, infection, or radiation, for instance) these cells proliferate and migrate to the target organ, where they produce high amounts of inflammatory cytokines leading to exacerbated inflammation (Figure 6).

Further studies must be conducted in order to evaluate how these DP-T cells are activated and whether they are antigen-specific or not. Although an increasing amount of evidences show the presence of extra-thymic DP-T cells in the control of inflammation [62]–[64], our data clearly shows that in malaria infection, the thymic-derived DP-T cells are inflammatory. To our knowledge, this is the first study that shows the interplay between malaria infection and autoimmune neuro-inflammation through the contribution of the thymus-derived double-positive T cells.

## Supporting Information

Figure S1 **Analysis of the gene expression in the thymus of mice infected with distinct** ***Plasmodium*** **species.** C57BL/6 mice were intraperitoneally (i.p.) infected with 1×10^6^ infected Red Blood Cells. Mice were infected with *P.berghei* NK65, *P.chabaudi* AS and *P.yoelli* CL. The gene expression of AIRE, FOXP3, Rorγt, IL-17, IL-7 and IL-6 was evaluated. Results showed that the different *Plasmodium* species triggers distinct gene expression. Data was analyzed by One-Way ANOVA and post-tested with Bonferroni, where *: p<0,05. Representative data of four independent experiments.
(TIF)

Figure S2 **Analysis of the inflammation in the CNS of malaria-cured EAE mice.** C57BL/6 mice were intraperitoneally (i.p.) infected with 1×10^6^ *P.berghei*-infected Red Blood Cells and treated with chloroquine (CQ, 5 mg/Kg) for five consecutive days starting at the 10^th^ day after infection. Three days after the last dose of CQ, mice were immunized with 100 µg of MOG_35–55_ peptide and Pertussis toxin was administrated (via i.p.) at 0 and 48 h after peptide immunization for EAE induction. The spinal cords of EAE mice were collected fourteen days after immunization. Frozen thin sections (12 µm) were made and fixed in formalin. Cells were stained with purified anti-mouse iNOS (in A), IL-6 (in B), COX-2 (in C), NF-κB (in D), phosphorylated iκBα (in E) and GFAP (in F). DAPI was added to stain DNA (blue color). The slices were analyzed in epifluorescence microscope. Figures are representative of three independent experiments. Magnification: 400X.
(TIF)
