# Supplementary figures and images for: Exacerbation of Autoimmune Neuro-Inflammation in Mice Cured from Blood-Stage Plasmodium berghei Infection
Source: PLoS One. 2014 Oct 17;9(10):e110739. doi: 10.1371/journal.pone.0110739 (PMC4201583; doi:10.1371/journal.pone.0110739)

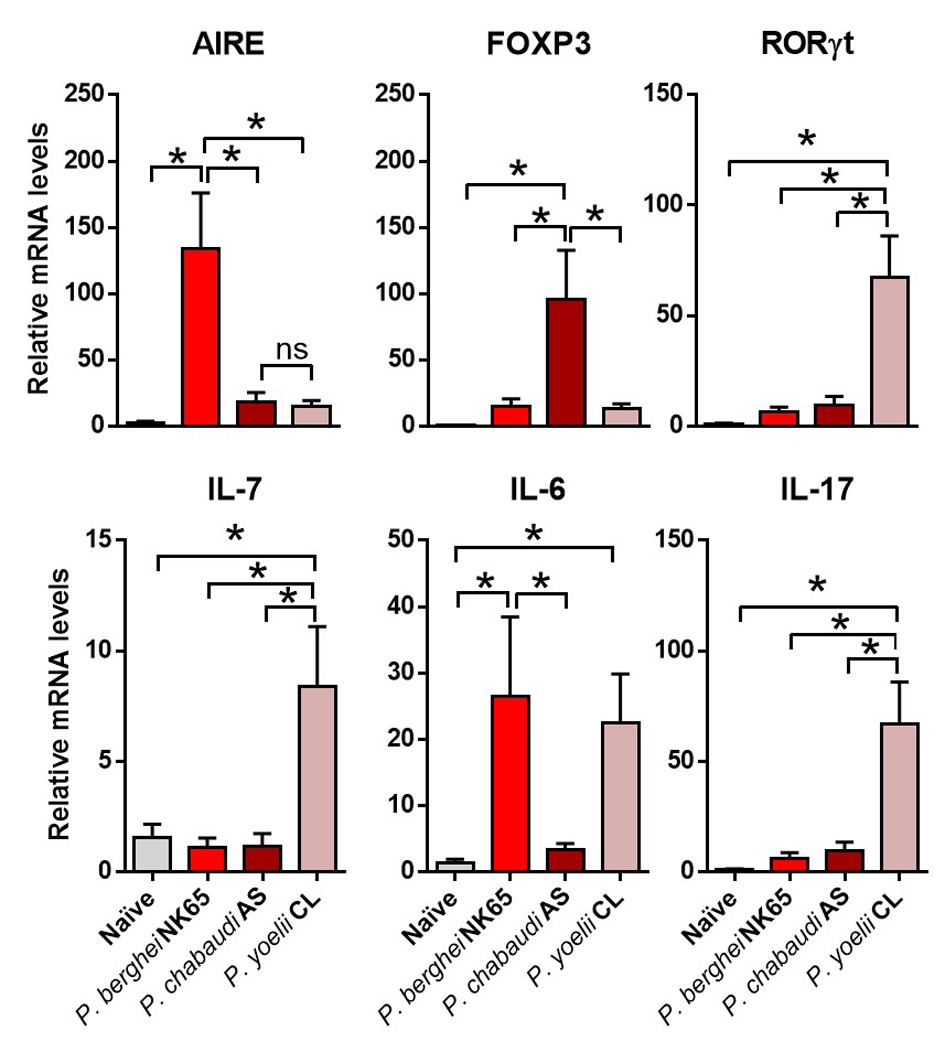

Supplement: Figure S1 — Analysis of the gene expression in the thymus of mice infected with distinct Plasmodium species. C57BL/6 mice were intraperitoneally (i.p.) infected with 1×10^6^ infected Red Blood Cells. Mice were infected with P.berghei NK65, P.chabaudi AS and P.yoelli CL. The gene expression of AIRE, FOXP3, Rorγt, IL-17, IL-7 and IL-6 was evaluated. Results showed that the different Plasmodium species triggers distinct gene expression. Data was analyzed by One-Way ANOVA and post-tested with Bonferroni, where *: p<0,05. Representative data of four independent experiments. (TIF) [file pone.0110739.s001.tif]

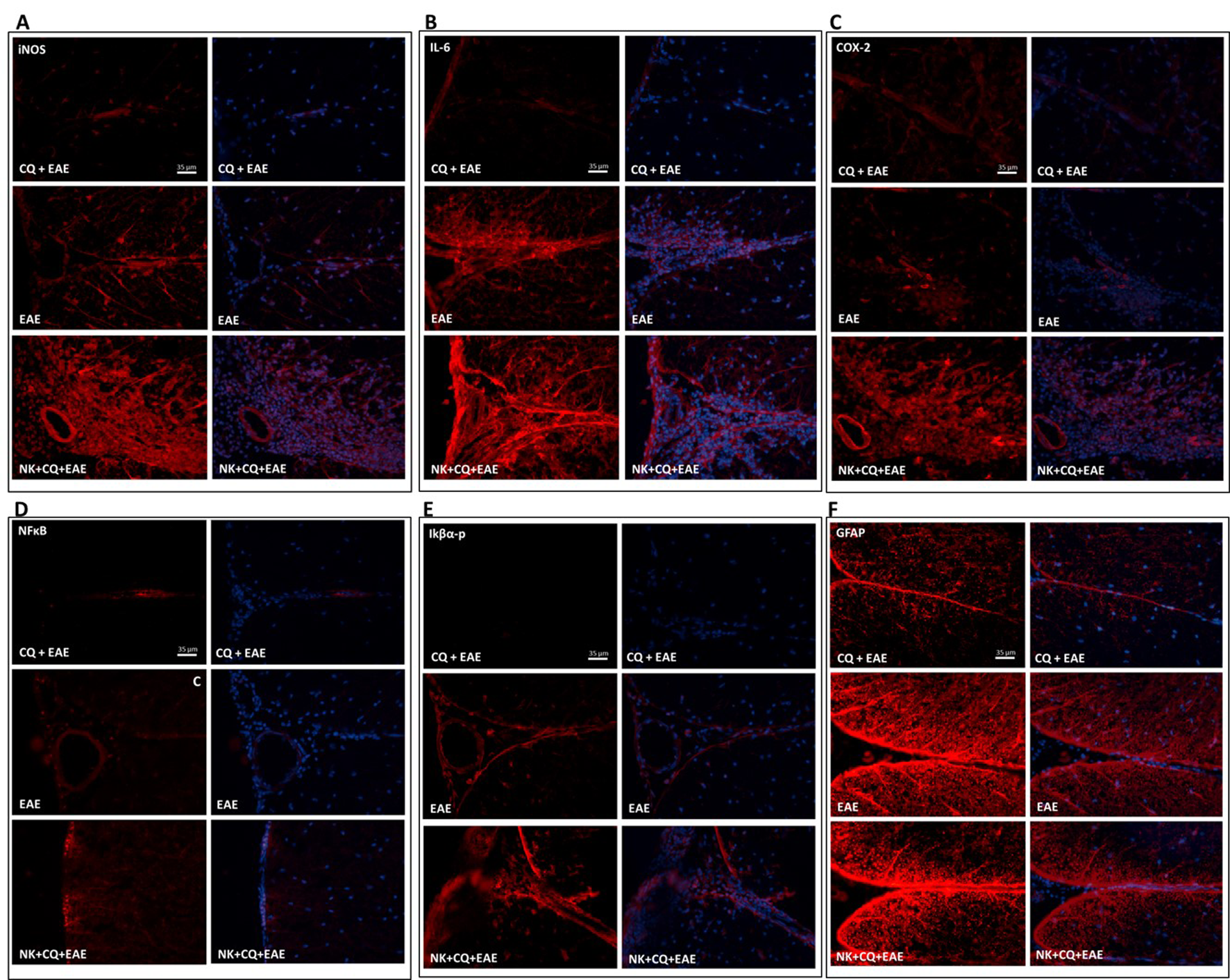

Supplement: Figure S2 — Analysis of the inflammation in the CNS of malaria-cured EAE mice. C57BL/6 mice were intraperitoneally (i.p.) infected with 1×10^6^ P.berghei-infected Red Blood Cells and treated with chloroquine (CQ, 5 mg/Kg) for five consecutive days starting at the 10th day after infection. Three days after the last dose of CQ, mice were immunized with 100 µg of MOG35–55 peptide and Pertussis toxin was administrated (via i.p.) at 0 and 48 h after peptide immunization for EAE induction. The spinal cords of EAE mice were collected fourteen days after immunization. Frozen thin sections (12 µm) were made and fixed in formalin. Cells were stained with purified anti-mouse iNOS (in A), IL-6 (in B), COX-2 (in C), NF-κB (in D), phosphorylated iκBα (in E) and GFAP (in F). DAPI was added to stain DNA (blue color). The slices were analyzed in epifluorescence microscope. Figures are representative of three independent experiments. Magnification: 400X. (TIF) [file pone.0110739.s002.tif]
